# Factors Associated with Failure of Bakri Balloon Tamponade for the Management of Postpartum Haemorrhage. Case Series Study and Systematic Review

**DOI:** 10.3390/healthcare9030295

**Published:** 2021-03-08

**Authors:** Francisco Javier Ruiz Labarta, María Pilar Pintado Recarte, Laura Joigneau Prieto, Coral Bravo Arribas, Julia Bujan, Miguel A. Ortega, Juan A. De León-Luis

**Affiliations:** 1 Department of Public and Maternal and Child Health, School of Medicine, Complutense University of Madrid, 28040 Madrid, Spain; javruila@hotmail.com (F.J.R.L.); ppintadorec@yahoo.es (M.P.P.R.); laurajoigneau@yahoo.es (L.J.P.); cbravoarribas@gmail.com (C.B.A.); jaleon@ucm.es (J.A.D.L.-L.); 2 Department of Obstetrics and Gynecology, University Hospital Gregorio Marañón, 28009 Madrid, Spain; 3 Health Research Institute Gregorio Marañón, 28009 Madrid, Spain; 4 Department of Medicine and Medical Specialties, Faculty of Medicine and Health Sciences, University of Alcalá, Alcalá de Henares, 28801 Madrid, Spain; mjulia.bujan@uah.es; 5 Ramón y Cajal Institute of Healthcare Research (IRYCIS), 28034 Madrid, Spain; 6 University Center for the Defense of Madrid (CUD-ACD), 28047 Madrid, Spain; 7 Pathological Anatomy Service, Central University Hospital of Defense-UAH, 28047 Madrid, Spain

**Keywords:** postpartum haemorrhage, Bakri balloon tamponade, failure factors

## Abstract

Background: Postpartum haemorrhage (PPH) is an unpredictable obstetric emergency that requires a multidisciplinary approach. Bakri balloon tamponade (BBT) is recommended when PPH does not respond to medical treatment. Nowadays few published studies have performed a multivariate analysis to determine the variables independently associated with BBT failure. Methods: Our study purpose was to determine the variables independently associated with BBT failure: first, in a large single-centre cohort study between 2010 and 2020, and second, in a systematic literature review using Medline and the Cochrane Library. Maternal and perinatal variables, PPH characteristics, technique-related variables and complications were recorded in the case series study, comparing between successful and failed BBT patients. Study characteristic and variables significantly associated with BBT failure were recorded in the systematic review. All studies used a logistic regression test. Results: The case series included 123 patients. The profile of these patients were primiparous, with vaginal delivery and a full-term new-born. BBT was successful in 81.3% of cases. Five studies were included in the systematic review, providing data from 551 patients. BBT was successful in 79.5% of cases. Conclusions: Maternal age, caesarean delivery, ≥7 red blood cells units (RBCU) transfused and curettage before BBT insertion, history of caesarean section, pre-pregnancy obesity, anteriorly placed placenta, placenta accreta, caesarean delivery, estimated blood loss before insertion of BBT, long operation duration, and coagulopathy were independent factors for BBT failure.

## 1. Introduction

Postpartum haemorrhage (PPH) is the leading cause of maternal mortality in low-income countries and the primary cause of nearly one-quarter of all maternal deaths globally [1]. Its incidence has increased in recent years, mainly due to increases twin pregnancies, labour induction, and caesarean sections [2,3,4], with up to 18% of births being complicated [5].

PPH is an obstetric emergency with an unpredictable and sudden onset that is associated with high maternal morbidity and mortality rates [6]. Thus, appropriate, and timely management is vital [7,8,9]. PPH requires a multidisciplinary approach with early aetiologic diagnosis, immediate control of blood loss, and patient stabilization.

The main cause of PPH is uterine atony [9], followed by retained placenta, placental abnormalities, genital tract laceration, and coagulopathies. Management depends on the aetiology and is dictated by several conditions, such as the desire to retain fertility and haemodynamic status [10,11]. First-line management for PPH includes conservative control with uterine massage, uterotonic drugs, surgical repair of genital tract lacerations, removal of retained placental tissues, vaginal packing, or correction of coagulation disorders. The active use of uterotonics in the third stage of labour is an essential component in the prevention and treatment of PPH [6,9]. Second-line management for PPH is still a challenge given the lack of controlled clinical trials [12]. When PPH does not respond to medical treatment, many guidelines recommend the use of an intrauterine balloon as a haemostatic tamponade method [13] before performing more invasive manoeuvres (uterine suture techniques [14], artery ligations [15] or, ultimately, emergency hysterectomy [16]) that are associated with loss of fertility and increased risk of infection, bleeding, or injury of adjacent organs.

During the last two decades, different uterine tamponade devices (Sengstaken–Blakemore tube, Foley, Rusch, or condom catheters) have been used successfully for the management of PPH [17]. Success rates for the control of PPH have ranged from 84% to 91% [13]. Bakri balloon tamponade (BBT) (Cook Medical, Spencer, IN, USA) is specifically designed for uterine tamponade in cases of acute PPH refractory to medical treatment, and it requires minimal training for correct use [18]. It is fast, highly effective, and safe and preserves fertility. Since it was first described in 2001 [19], case series have reported high clinical success rates of BBT in PPH with good clinical outcomes, and various studies have analysed the risk factors associated with balloon failure. However, very few studies have performed a multivariate analysis that weights the effect of each factor in the presence of the others [20,21,22,23,24].

The purpose of this study was to determine the variables associated with the failure of BBT for the management of PPH. To this end, we performed an analysis of the clinical outcomes of BBT in a large single-centre cohort study. Secondly, we conducted a systematic review of the literature to search for studies that performed a multivariate analysis to determine the variables independently associated with BBT failure.

## 2. Materials and Methods

### 2.1. Case Series of BBT for PPH

A prospective cohort observational study was carried out in the obstetrics and gynaecology unit of our tertiary referral centre in Madrid (Spain). All consecutive women with PPH who underwent BBT between 1 January 2010 and 15 May 2020 were included. The Institutional Review Board approved our study protocol (HOS), and verbal informed consent was obtained from all patients because of the urgent nature of the procedure.

In our centre, we define PPH as an estimated blood loss greater than 500 mL after a vaginal delivery and greater than 1000 mL after a caesarean section [8]. We used a collection bag after vaginal delivery to reliably estimate blood loss and pre-weighed the textile material. During caesarean section, blood loss is estimated using a surgical aspirator [25]. We always consider the patient’s clinical condition in relation to the degree of shock.

Before BBT placement, the patients were clinically examined and were treated for PPH according to our institutional protocol. As first-line management for PPH, it includes staggered use of uterotonic drugs (first oxitocine perfusion, second intramuscular methylergometrine, and third intrarectal misoprostol). The patients were managed by a multidisciplinary team comprising obstetrics and anaesthesiology staff.

BBT insertion is performed in the operating room by different gynaecologists in our unit. The technique used to apply the device is similar to the original technique described by Bakri et al. [19]. The balloon is inserted transvaginally (in most cases) or transabdominally (in some cases of caesarean section). The balloon is filled with a certain amount of saline depending on the size and capacity of the uterus based on the point of resistance during infusion. Vaginal packing around the balloon shaft is applied in all cases. After placement, medical treatment includes oxytocin 10 IU in 500 mL normal saline at a rate of 125 mL/h by continuous perfusion and 2 g of cefazolin as broad-spectrum antibiotic prophylaxis. All patients have a Foley catheter inserted for urine output monitoring. Transabdominal ultrasonographic scans are routinely performed to check the balloon position. After Bakri balloon placement, patients are transferred to the post-anaesthesia recovery unit (PARU) or to the intensive care unit (ICU) for constant surveillance with control of hemogram, arterial pressure and cardiac frequency for 6–12 h). The BBT is removed at the discretion of the gynaecologist, usually in two stages, after it has been in place for at least 12 h.

Patients for whom first-line management failed to stop PPH and who were managed with intrauterine BBT were included in the present study. The BBT cases were included in an electronic database for subsequent analysis. Pre-procedure assessment involved the collection of clinical maternal and perinatal characteristics, including maternal age, parity, previous caesarean section, type of gestation (singleton or multiple), gestational age at delivery, induction of labour, type of delivery (vaginal or caesarean section), and neonatal birth weight (g). The PPH characteristics and maternal haemodynamic state data that were collected included type of PPH (primary (<24 h post-partum) or secondary (between 24 h and 6 weeks after delivery)), aetiology of PPH, pre-, and post-BBT surgical procedures (curettage, bilateral uterine artery ligation, B-Lynch suture, and obstetric hysterectomy), need for transfusion (mean number of red blood cell units (RBCUs) and fresh-frozen plasma units (FFPUs) per patient) and the presence of disseminated intravascular coagulation (DIC). BBT main parameters were recorded, including BBT placement route, balloon output, filling volume and duration of placement. We collected and analysed the antepartum and intrapartum risk factors that are most frequently cited in the literature [26].

BBT success was defined as complete haemorrhage arrest with haemodynamic stability and no need for a subsequent surgical procedure or pelvic arterial embolization (PAE). The surgical procedures used after BBT failure and complications were recorded.

Statistical analysis was performed using the SPSS software package, version 25 (IBM Co., Somers, NY, USA), with its default settings. Outcomes were compared between successful and failed BBT for PPH. Quantitative variables are expressed as the mean ± standard deviation; qualitative variables are expressed as numbers (percentages). The Mann–Whitney U-test or Wilcoxon test was used to compare the median values of quantitative variables, and Fisher’s exact test or the chi-square test was used to compare qualitative variables. *p* < 0.05 was accepted as statistically significant. BBT failure factors with *p* < 0.2 or clinical relevance in the univariate analysis were tested in the multivariate analysis using a binary logistic regression test. Odds ratios (ORs) with 95% confidence intervals (CIs) were calculated.

### 2.2. Systematic Review

The PRISMA statement [27] was followed in this review. The study was registered with the PROSPERO database (registration number: CRD42018116528).

### 2.3. Eligibility Criteria and Outcome Measures

All observational cohort studies of BBT to treat PPH that reported a multivariate analysis using a logistic regression test were included in this systematic review. The primary outcome was to identify factors significantly associated with failure of BBT.

### 2.4. Information Sources and Search Strategies

Searches were conducted across international electronic bibliographic databases (Medline and Cochrane Library) to identify studies published until 15 May 2020. We did not exclude any articles because of their language. A reference database (EndNote X7, Thomson Reuters, New York, NY, USA) was used to incorporate all references.

Articles were identified using comprehensive search criteria and a combination of MeSH terms with the following keywords: “postpartum haemorrhage” or “postpartum haemorrhage” and “Bakri balloon” or “Bakri balloon tamponade” or “Bakri SOS balloon” or “Bakri surgical obstetric silicone balloon”.

### 2.5. Study Selection, Data Extraction, Statistical Analysis, and Risk of Bias

Two independent researchers (RL, LL) analysed the titles and abstracts obtained to select relevant articles. If the title and abstract did not provide sufficient information, the full text was retrieved. Duplicates, letters to editors, editorials, and review articles were excluded. 

The studies included in this review were selected by both authors after independent application of the eligibility criteria. Disagreement between the two researchers was resolved by consensus.

A data extraction sheet was completed with the variables studied: author; publication year; country; study characteristics; number of cases (*n*); aetiology of PPH; rate of vaginal/caesarean delivery; rate of success, complications and factors significantly associated with failure or success of BBT. Each reviewer collected the data independently and included them in the extraction sheet. Discrepancies were resolved by both authors checking the study against the form.

Success of BBT was defined as complete haemorrhage arrest with haemodynamic stabilization and no subsequent surgical procedure or embolization.

Statistical analysis was performed using SPSS Version 21.0 (IBM Corp.) with its default settings. We attempted to carry out a quantitative synthesis with pooled relative risks and 95% CIs, but a meta-analysis was not feasible given the lack of a control group and heterogeneity of available studies.

The risk of bias was assessed by both authors independently determining the adequacy of compliance with the inclusion criteria. Items assessed were consecutive recruitment, correct description of cases included, procedures undertaken, and complete reporting of outcomes and complications. Any disagreements were resolved by discussion. The quality of the evidence of the included studies was assessed according to the Grade of Evidence Working Group Criteria [28].

## 3. Results

### 3.1. Case Series of BBT for PPH

During the 10-year study period, there were 53,977 deliveries at our centre. A total of 123 women (0.23% of all deliveries) underwent BBT insertion for PPH after unsuccessful first-line management.

The profile of patients who underwent BBT placement is primiparity (65.9%), age 33.7 years, Spanish origin (56.9%), with vaginal delivery (57.7%), and a full-term new-born (71.5%). In such patients, labour was induced with prostaglandin in 47.2% of cases, and 22% of pregnancies were multiple gestations (all twin pregnancies). A total of 12.2% of patients had had a previous caesarean section delivery (Table 1). 

The most frequently occurring risk factors for antepartum bleeding in our series were multiple gestation and placenta previa. A total of 39.8% of patients had no antepartum risk factors. Among the intrapartum risk factors, emergency caesarean section, labour induction, and instrumental delivery stood out.

The majority of PPHs occurred in the first 24 h after delivery (93.5%), and the main indication for BBT was uterine atony (69.6%), followed by retention of placental fragments (11.4%). In 70.7% of cases, blood loss was estimated at 1000–2500 mL. A total of 69.1% of the patients required transfusion of packed red blood cells, and the patients received a mean of 5.25 units of packed red blood cells (range: 0–40).

Many patients (65%) required uterine curettage prior to BBT placement. The BBT was inserted vaginally in 95.1% of cases, and a mean of 255 mL of saline was used to inflate the BBT. The BBT was left in utero for 18.7 h, and 173 mL of blood was drained while the balloon and catheter were in place. A total of 82.1% of the patients required admission to the PARU and 17.1% required admission to the ICU, with a median length of hospital stay of 5.2 days.

**Figure 1:**
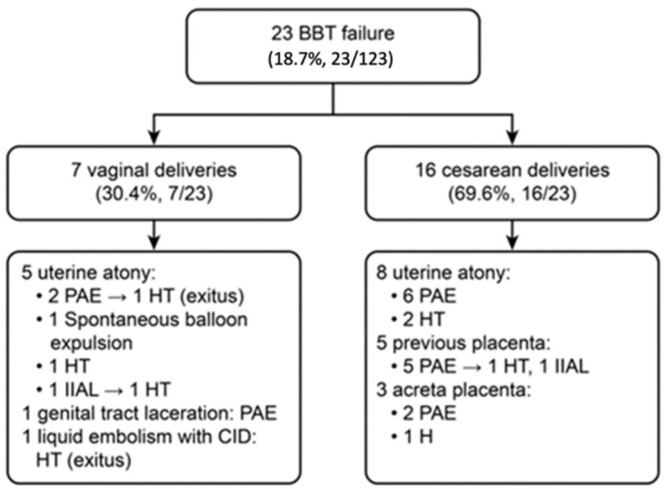
Flowchart of studies retrieved and included in the systematic review. Abbreviation: BBT: Bakri Balloon Tamponade. PAE: Pelvic Arterial Embolization. HT: Hysterectomy. IIAL: Internal Iliac Arterial Ligation. DIC: disseminated intravascular coagulation.

PPH was controlled with BBT in 100 (81.3%) cases. It was effective in 36 (69.2%) cases of PPH after caesarean delivery and in 64 (90.1%) cases of PPH after vaginal delivery (*p* < 0.05). There were 23 (18.7%) cases of BBT failure, and these patients received additional treatment because of insufficient haemostasis (16 PAEs, 2 vascular ligations, and 8 puerperal hysterectomies) (Figure 1). In one case, spontaneous expulsion of the BBT occurred immediately after insertion and filling. Fortunately, haemostasis was achieved, and other procedures were not needed.

Univariate analysis (Table 1) showed that patients were more likely to have BBT failure if they were younger, had a lower haematocrit value on admission, were of Asian origin, had placenta previa, had a caesarean delivery or had an estimated blood loss before BBT placement >1000 mL (*p* < 0.05). The mean number of RBCUs, platelets and FFPUs transfused was higher in the BBT failure group (*p* < 0.05). The clinical success of BBT was related to curettage before BBT insertion, vaginal insertion, and duration of in utero placement (*p* < 0.05). 

Multiple regression analysis (Table 2) revealed that maternal age, caesarean delivery, ≥7 RBCUs transfused and curettage before BBT insertion were variables significantly and independently associated with BBT failure.

There were no major complications arising from the use of the BBT. Two patients died. One patient had hypovolemic shock, coagulopathy, and multiorgan failure after PPH due to uterine atony, despite invasive management to control bleeding. Another patient died due to amniotic fluid embolism with DIC and massive bleeding.

### 3.2. Systematic Review

**Figure 2:**
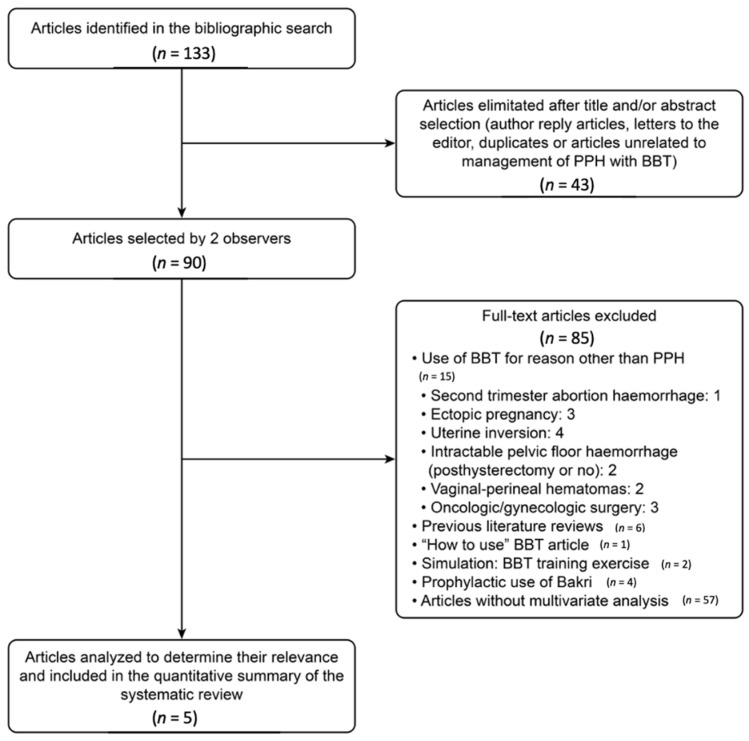
Flowchart to describe posterior management of Bakri Balloon Tamponade (BBT) failure cases.

A total of 133 citations were identified in the bibliographic search. The initial screening identified 43 articles as duplicates, letters, or editorials or as unrelated to the management of PPH with BBT. Of a total of 90 studies that were retrieved to assess eligibility, five were selected as relevant [20,21,22,23,24]. A flowchart for study selection is presented in Figure 2.

All five studies were observational cohort studies, and they presented a total of 551 patients. Three studies were multicentre. Table 3 shows the characteristics of the included studies and the main results extracted.

The articles analysed were not homogeneous in terms of the assessment of blood loss and PPH aetiology. The largest study (including 10 maternity units), by Revert et al. [20] (*n* = 226), reported a success rate of 83.2%, and Cho et al. [22] reported the smallest number of cases (*n* = 64), with a success rate of 75%. The most common cause of PPH was uterine atony (50%), followed by placenta previa (28.1%). Two studies only included patients with placenta previa and caesarean delivery (21–22), and 1 study that only included patients with vaginal delivery [24]. The global success rate for BBT was 79.5%.

The variables significantly associated with BBT failure (multivariate analysis) were history of caesarean section, pre-pregnancy obesity, anteriorly placed placenta, placenta accreta, caesarean delivery, estimated blood loss before insertion of BBT, long operation duration, and coagulopathy (Table 4).

The emergency hysterectomy rate was 6%, and the mortality rate was 0.2% (1 patient). Only one case of endometritis was reported, and 2 patients had thromboembolic events.

Given the type of articles included (observational cohort studies without a control group), an assessment of bias was difficult to perform.

## 4. Discussion

### 4.1. Main Findings

#### 4.1.1. Case Series of BBT for PPH

Our prospective cohort study revealed that maternal age, caesarean delivery, ≥7 RBCUs transfused, and curettage before BBT insertion are variables directly, significantly, and independently associated with BBT failure. Maternal age may be a reason for BBT failure because advanced age is well known as a risk factor of PPH and other intrapartum complications [3,26]; additionally, older patients have a greater number of comorbidities than younger patients. Caesarean delivery has previously been identified as a variable associated with BBT failure [20]. This may be because it may be more difficult to control bleeding because the uterus has a scar, has a greater tendency towards atony and bleeds more and because it takes longer to place the BBT than during a vaginal delivery. Additionally, the underlying causes/indications for caesarean section such as placenta previa, no labour progression, twin pregnancy, pelvic-cephalic disproportion due to foetal macrosomia are all of them well known risk factors for PPH. Transfusion of ≥7 RBCUs is associated with BBT failure because in such situations, there is late action, and the patient has lost a large volume of blood before BBT placement and may be in a state of coagulopathy, which are factors previously associated with BBT failure in other studies [20,23]. Finally, we assume that curettage before BBT insertion is associated with BBT failure because it may delay the early placement of the device, thus increasing the amount of bleeding. In addition, the most frequent cause of PPH is atony, and curettage is not necessary in such cases because it is only used for the extraction of retained ovular fragments. There is strong evidence suggesting that a prolonged time between onset of haemorrhage and placement of uterine balloon tamponade results in worse outcomes [29,30].

BBT is effective and safe for the management of PPH. According to previously published reviews [31], the clinical success rate is approximately 85.9%, and the frequency of complications attributed to device use is low (≤6.5%). The success rate in our series of patients was 81.3% based on criteria used in published studies. Complications after BBT placement have been described in the literature and include fever, endometritis, uterine necrosis, cervical tears, scar dehiscence, or uterine perforation [31]. In our series of patients, there were no complications.

At our centre, BBT was used most frequently in primiparous women (65.9%), after the induction of labour (47.2%), and in cases of vaginal delivery (57.7%) for PPH due to uterine atony (69.6%). Its more frequent use in primiparous women could be related to the fact that BBT is a fertility-sparing technique. Compared to the profile of pregnant women who attended our hospital during the same period, there was a significantly higher rate of twin pregnancy (22% vs. 3%; *p* < 0.001) and labour induction (47.2% vs. 20%; *p* < 0.001) among BBT cases. These variables have been previously associated with PPH due to uterine atony in other centres [32,33]. The caesarean section rate among patients undergoing BBT insertion was 42.3%, which is much higher than the caesarean rate of the general population (18% in our centre). This could be explained by the fact that most of the risk factors for PPH are also indications for caesarean delivery (TOPP, for instance) and by the fact that the caesarean section itself is a risk factor for PPH.

Compared with the literature reported, our series of patients had a higher rate of PAE after the failure of BBT (13%) and a lower rate of puerperal hysterectomies (6.5%). Danisman et al. [34] reported a hysterectomy rate of 50%, Cekmez et al. [35] reported a rate of 20%, and Kaya et al. [32] reported a rate of 11%. The difference may be because our centre used an interventional technique that is not available in many centres, and we managed to treat the haemorrhage quickly, before the patient reached haemodynamic instability. Our working group published an article about outcomes of PAE in the management of PPH [11], including 1739 patients. PAE was successful in 89.4% of cases and complications were uncommon (1.8%). According to our data and other published articles [13], the success rate of PAE and BBT appears to be similar, with low complication rate. BBT is the first step in the management of PPH in our center because it is a less invasive and quicker approach. PAE requires a vascular and interventional radiology ward where the patient must be transferred to, while BBT is easy to assemble and can be inserted in the delivery or operating room.

Ramler et al. [36] performed a study to compare the outcomes of intrauterine balloon tamponade and uterine artery embolization for persistent postpartum haemorrhage (defined as PPH refractory to first-line treatment combined with at least one uterotonic agent). They found there was no significant difference in the risk of post-partum hysterectomy and/or maternal death, total volume of blood loss, or total number of packed red blood cells between the two groups. However, the small size of the sample did not allow to determine whether intrauterine balloon tamponade is equivalent or superior to other management strategies and larger studies are necessary.

#### 4.1.2. Systematic Review

In this systematic review, we found that the variables associated with BBT failure were history of caesarean section, pre-pregnancy obesity, anteriorly placed placenta, placenta accreta, caesarean delivery, estimated blood loss before insertion of the BBT, long operation duration, and coagulopathy. Previous caesarean section, obesity, caesarean delivery, and placenta accreta are well established as risk factors for PPH in previous studies [26]. High blood loss prior to BBT insertion, long operation duration, and coagulopathy are variables that are surely associated with late action in PPH. A question that needs to be addressed in the guidelines is the recommended timepoint for BBT use. In light of the results of this review, given the high success rate and device safety, we can conclude that BBT should be used as soon as pharmacological treatment seems to be failing. Studies are being undertaken to assess the usefulness of early placement of BBT as first-line therapy, together with prostaglandin administration [31]. BBT could also have a role as temporary treatment to minimize blood loss in patients who need to be transferred to an interventional radiology department to undergo PAE.

#### 4.1.3. Strengths and Limitations

Our study presents the first systematic review of the literature on variables associated with BBT failure and contributes the results obtained from the multivariate analysis of our case series. In the multivariate analysis of our series of patients, we found three variables associated with BBT failure (maternal age, ≥7 units of packed red blood cells transfused and curettage before BBT insertion) that has never been reported in the current literature. In particular, the difference we found in the effect of curettage on BBT failure (a positive effect in the univariate analysis and a negative effect in the multivariate analysis) highlights the importance of multivalent analysis. This analysis allows the study of the influence of one variable in the presence of other risk factors, which allowed us to control the confounding effect that each variable can mask when analysed independently.

Many case series have evaluated the use of BBT for the conservative treatment of PPH, but they included small numbers of patients. Our report includes one of the largest series of patients (*n* = 123) published in the literature and collected from a single centre. The study with the largest sample size published to date was reported by Wang et al. [29] in 2018 and included 407 patients in a prospective observational multicentre cohort study.

Our study has some limitations, such as its observational nature and lack of a comparison group. A randomized study is difficult to design and carry out because of the emergency context in which treatment decisions need to be made and the lack of appropriate resources at many centers. The study period was long (10 years), and the results may be somewhat affected by this duration because of our increasing use of BBT and the increasingly early placement of balloons, a factor that may not have happened in our first years of applying this treatment. Therefore, the publication of large case series from recent years is necessary to contribute to the validity of our results. Results may be biases from unknown factors that have been not collected in our study, even though exhaustive data were collected for all important factors potentially associated with BBT failure.

It is acknowledged that review articles are conditioned by the quality of the publications they summarize. Given that all the included reports were observational case series studies, the quality of the evidence is graded low/very low according to the Grade of Evidence Working Group Criteria [28]. The results should be interpreted with caution because the confidence intervals were very wide due to the small number of cases in the BBT failure group.

We are aware of the fact that there could be selection bias as well as publication bias due to incomplete reporting or non-reporting of cases with low success rates or high complication rates. Randomized trials would allow us to control these biases.

## 5. Conclusions

Both the prospective cohort study and the systematic review revealed that the variables associated with BBT failure are history of caesarean section, pre-pregnancy obesity, maternal age, anteriorly placed placenta, placenta accreta, caesarean delivery, ≥7 RBCUs transfused, curettage before BBT insertion, estimated blood loss before BBT insertion, long operation duration, and coagulopathy. The information gained from this study is helpful in counselling obstetricians to managing PPH cases better. Women with several of these factors are high-risk women for failed BBT and will probably require more often invasive procedure (surgery or embolization) to achieve bleeding and to improve maternal outcomes. 

It would be interesting to create an international registry of cases in which data about patients treated with BBT are cumulatively collected. This would probably provide some answers to the problems we currently face (i.e., measuring the severity of PPH, evaluating the efficacy of treatment, and detailing its complications).

## Figures and Tables

**Table 1:** Clinical characteristics of women managed with Bakri Balloon Tamponade (BBT) for postpartum haemorrhage (PPH) in the study hospital (*n* = 123). Comparison between successful and failed management with BBT.

Characteristics	Total (*n* = 123)	BBT Success Group (*n* = 100)	BBT Failure Group (*n* = 23)	*p* Value
Maternal and perinatal characteristics				
Maternal age (years)	33.7 ± 5.7 (21–45)	34.3 ± 5.3 (21–45)	31.1 ± 6.7 (21–43)	0.043
Primiparous	81 (65.9%)	63 (63%)	18 (78.3%)	0.164
History of caesarean section	15 (12.2%)	11 (11%)	4 (17.4%)	0.398
Twin pregnancy	27 (22%)	24 (24%)	3 (13%)	0.252
Gestational age at delivery (weeks)	37.4 ± 3.3 (24–42)	37.3 ± 3.4 (24–41)	37.7 ± 2.6 (31–42)	0.898
hematocrit on admission (%)	35.2 ± 4.1 (22.1–43.9)	35.6 ± 4.2 (22.1–43.9)	33.9 ± 2.9 (26.9–37.5)	0.019
Induction of labour	58 (47.2%)	50 (50%)	8 (34.8%)	0.187
Caesarean delivery	52 (42.3%)	36 (36%)	16 (69.6%)	0.003
Neonatal birth weight (g)	2883.2 ± 758.4 (600–4320)	2851.2 ± 794.5 (600–4320)	3022.2 ± 569.7 (1820–4020)	0.431
PPH characteristics and maternal haemodynamic state data				
Primary PPH	115 (93.5%)	93 (93%)	22 (95.7%)	0.642
Aetiology of PPH				
Uterine atony	86 (69.9%)	73 (73%)	13 (56.5%)	
Retention of placental fragments	14 (11.4%)	14 (14%)	0	
Placenta previa	6 (4.9%)	1 (1%)	5 (21.7%)	
Placenta accreta	8 (6.5%)	5 (5%)	3 (13%)	
Vaginal/cervical tears	3 (2.4%)	2 (2%)	1 (4.3%)	
DIC	2 (1.6%)	1 (1%)	1 (4.3%)	0.001
Curettage before BBT insertion	80 (65%)	71 (71%)	9 (39.1%)	0.004
Estimated blood loss before BBT				
1000 mL	23 (18.7%)	21 (21%)	2 (8.7%)	
1000–2500 mL	87 (70.7%)	75 (75%)	12 (52.2%)	
2500–5000 ml	13 (10.6%)	4 (4%)	9 (39.9%)	0.001
First post-bleed hematocrit (%)	25.6 ± 5.2 (11.8–38.7)	25.6 ± 5.3 (14–38.7)	25.2 ± 4.6 (11.8–31.6)	0.984
Need for transfusion	85 (69.1%)	64 (64%)	21 (91.3%)	0.011
RBCUs per patient	5.2 ± 5.4 (0–40)	3.4 ± 2.1 (0–11)	11.1 ± 7.9 (1–40)	0.001
≥7 RBCUs transfused	21 (17.1%)	5 (5%)	16 (69.6%)	0.001
FFPUs per patient	3.7 ± 4.9 (0–30)	1.9 ± 1.4 (0–4)	5.8 ± 6.4 (0–30)	0.001
Number of concentrated platelets transfused per patient	1.7 ± 2.6 (0–14)	0.6 ± 0.6 (0–2)	2.6 ± 3.4 (0–14)	0.002
Fibrinógeno (gr) per patient	2.9 ± 2.7 (0–20)	2.1 ± 0.9 (0–6)	4.8 ± 4.4 (1–20)	0.001
Admission to PARU	101 (82.1%)	98 (98%)	3 (13%)	0.001
Admission to ICU	21 (17.1%)	2 (2%)	19 (82.6%)	0.001
Hospital stay (days)	5.2 ± 5.3 (1–49)	4.4 ± 3.3 (2–25)	9.1 ± 9.3 (1–49)	0.001
BBT main parameters				
Vaginal BBT placement	117 (95.1%)	97 (97%)	20 (87%)	0.044
Filling volumen (mL)	255.6 ± 100.7 (60–540)	250.3 ± 92 (90–500)	286.5 ± 141.3 (60–540)	0.435
Balloon output (mL)	173.4 ± 264.5 (0–1800)	109.5 ± 89.9 (0–700)	573.1 ± 534.3 (25–1800)	0.001
Duration of placement (hours)	18.7 ± 8.1 (0–36)	20.4 ± 5.8 (2–36)	11.5 ± 12.1 (0–36)	0.003

Values are given as mean ± SD (range) or number (percentage). Abbreviation: BBT: Bakri Balloon Tamponade. PPH: Postpartum Haemorrhage. SD: standard deviation. PARU: post-anaesthesia recovery unit. ICU: intensive care unit. RBCUs: red blood cell units. FFPUs: fresh-frozen plasma units. DIC: disseminated intravascular coagulation.

**Table 2:** Logistic regression analysis of factors for failed Bakri Balloon Tamponade (BBT) management.

Variables Significantly Associated with Failed BBT	OR	95% CI	*p* Value
Maternal age	1.26	1.07–1.47	0.01
Caesarean delivery	6.90	1.23–38.65	0.03
Curettage before BBT insertion	9.02	1.69–48.22	0.01
≥7 RBCUs transfused	68.39	12.60–371.33	<0.001

Abbreviation: BBT: Bakri Balloon Tamponade. OR: Odds Ratio. CI: Confidence interval. RBCU: Red Blood Cell Units.

**Table 3:** Characteristics and clinical results of studies reported in the systematic review.

Report (Author and Year)	Country	Type of Study	*n*	Vaginal/Caesarean Delivery *n* (%)	Indications for BBT *n* (%)	Clinical Success *n* (%)	Hysterectomy *n* (%)	Death *n* (%)	BBT-Related Complications
Revert M et al. 2016 [20]	France	Prospective cohort study; 10 maternity units	226	171 (75.5%)/55 (24.3%)	Uterine atony: 183 (81%); Placenta previa: 33 (14.6%); Others: 10 (4.4%)	188 (83.2%)	11 (4.9%)	0	1 endometritis
Maher MA et al. 2017 [21]	Saudi Arabia	Prospective cohort study; 2 hospitals	72	Cesarean: 72 (100%)	Placenta low-lying: 42 (58.5%); Placenta incomplete centralis: 20 (27.7%); Placenta complete centralis: 10 (13.8%)	63 (87.5%)	1 (1.4%)	0	0
Kong CW et al. 2018 [23]	Hong Kong	Retrospective	81	24 (29.6%)/57 (70.4%)	Uterine atony: 53 (65.4%); Placenta previa/accreta: 25 (30.9%); Uterine/vaginal/cervical tears: 3 (3.7%)	59 (72.8%)	11 (13.6%)	1 (1.2%)	0
Cho HY et al. 2015 [22]	Korea	Retrospective	64	Cesarean: 64 (100%)	Placenta previa totalis: 50 (78%); Placenta previa partialis. marginalis: 11 (17%); Low-lying placenta: 3 (5%)	48 (75%)	5 (8%)	0	0
Grange J et al. 2018 [24]	France	Retrospective case series study; 5 maternity units	108	Vaginal: 108 (100%)	Uterine atony: 39 (36.1%); Placenta previa: 6 (5.5%); Placenta accreta: 3 (2.8%); Retained placenta: 23 (21.3%)	80 (74.1%)	5 (4.6%)	0	2 thromboembolic events
Total			551	303 (55%)/248 (45%)	Uterine atony: 275 (50%); Placenta previa: 155 (28.1%)	438 (79.5%)	33 (6%)	1 (0.2%)	

Values are given as mean ± SD or number (percentage). Abbreviation: BBT: Bakri balloon Tamponade.

**Table 4:** Logistic regression analysis of studies reported in the systematic review.

**Report** **(Author and Year)**	**Variables Significantly Associated with Failed BBT**	**OR**	**95% CI**	***p* Value**
Revert M et al. 2016 [20]	Caesarean delivery	3.5	1.6–7.6	<0.05
	Estimated blood loss before BBT	3.2	1.5–6.8	<0.05
	Coagulopathy	5.6	2.5–13.0	<0.05
Cho HY et al. 2015 [22]	Anterior placenta	12.75	1.04–155.94	0.04
	History of cesarean section	8.9	2.27–34.83	<0.01
Grange J et al. 2018 [24]	Pre-pregnancy obesity	4.4	1.06–18.31	<0.05
**Report** **(Author and Year)**	**Variables Significantly Associated with Successful BBT**	**OR**	**95% CI**	***p* Value**
Maher MA et al. 2017 [21]	Placenta accreta	0.01	0.00–0.97	0.049
	Operation duration (min)	1.14	1.02–1.28	0.023
Kong CW et al. 2018 [23]	Blood loss at the time of insertion of BBT	0.99	0.99–0.99	0.041
	Volume of blood drained from the uterine cavity within first 30 min	0.97	0.95–0.99	0.034
	Placenta accreta	0.01	0.01–0.98	0.048
	Coagulopathy	0.02	0.01–0.96	0.048

Abbreviation: BBT: Bakri Balloon Tamponade. OR: Odds Ratio. CI: Confidence interval.

## Data Availability

The data used to support the findings of the present study are available from the corresponding author upon request.
